# Pygo2 as a novel biomarker in gastric cancer for monitoring drug resistance by upregulating MDR1

**DOI:** 10.7150/jca.53356

**Published:** 2021-03-15

**Authors:** Dongdong Zhang, Yu Liu, Qiuwan Wu, Yahong Zheng, Natasha Mupeta Kaweme, Zhiming Zhang, Mingquan Cai, Youhong Dong

**Affiliations:** 1 Department of Oncology, Xiangyang No. 1 People's Hospital, Hubei University of Medicine, Xiangyang, Hubei 441000, China.; 2 Department of Medical Oncology, Xiamen Key Laboratory of Antitumor Drug Transformation Research, The First Affiliated Hospital of Xiamen University, School of clinical Medicine, Fujian Medical University, Xiamen 361003, Fujian Province, P.R. China.; 3 Xiamen Huli District Maternal and Child Health Hospital, 361005 Xiamen, Fujian, China.; 4 Department of Hematology, Zhongnan Hospital, Wuhan University, Wuhan, P.R. China.

**Keywords:** Pygo2, gastric cancer, biomarker, drug resistance, MDR1

## Abstract

Chemotherapy is the main therapy for gastric cancer (GC) both before and after surgery, but the emergence of multidrug resistance (MDR) often leads to disease progression and recurrence. P-glycoprotein, encoded by *MDR1*, is a well-known multidrug efflux transporter involved in drug resistance development. Pygo2 overexpression has been identified in several cancers. Previous studies have shown that abnormal expression of Pygo2 is related to tumorigenesis, chemoresistance, and tumor progression. In this study, to evaluate the underlying relationship between Pygo2 and MDR1 in GC, we constructed GC drug-resistant cell lines, SGC7901/cis-platinum (DDP), and collected tissue from GC patients' pre-and post-chemotherapy. We found that Pygo2 was overexpressed in GC, especially in GC drug-resistant cell lines and GC patients who underwent neoadjuvant DDP-based chemotherapy. Pygo2 overexpression may precede MDR1 and correlates with MDR1 in GC patients. Furthermore, knock-down of Pygo2 induced downregulation of MDR1 and restored SGC7901/DDP's sensitivity to DDP. Further mechanistic analysis demonstrated that Pygo2 could modulate MDR1 transcription by binding to the MDR1 promoter region and promoting MDR1 activation. The overall findings reveal that Pygo2 may be a promising biomarker for monitoring drug resistance in GC by regulating MDR1.

## Introduction

Gastric cancer (GC) has relatively high morbidity and mortality rate around the world. Although surgery is the first choice of treatment, chemotherapy also plays an important role in treating GC as GC has a low incidence of early diagnosis. Neoadjuvant chemotherapy can diminish tumor volume, degrade tumor stage, and increase the resection rate. Postoperative chemotherapy can prevent tumor recurrence and metastasis, thus prolonging the overall survival. Palliative chemotherapy can alleviate clinical symptoms and improve the quality of life of patients. However, multidrug resistance (MDR) can eventually result in chemotherapy failure. Therefore, early detection of MDR in GC patient is essential.

Cancer cells can pump various chemotherapeutic drugs out of the cell by drug efflux transporters 1. The occurrence of MDR is accompanied by the overexpression of multidrug efflux transporters, with the most extensively studied being multidrug resistance protein 1 (MDR1) 2. P-glycoprotein, encoded by ATP-binding cassette subfamily B member 1 (ABCB1) or *MDR1*, can induce multidrug resistance by increasing drug efflux and decreasing intracellular drug concentration 3. The overexpression of MDR1 was identified in some intrinsically drug-resistant digestive system tumors such as colorectal carcinoma, hepatocellular carcinoma, pancreatic cancer 4, 5, and other tumors 2. Furthermore, MDR1 overexpression in breast cancer often indicated a more malignant phenotype 6 and a higher recurrence rate 7. To date, the molecular modulation mechanism of MDR1 in gastric cancer is still unclear.

Pygopus2 (Pygo2) was initially identified as a novel functional protein of the Wingless pathway downstream in *Drosophila* 8. Further studies showed Pygo2 could regulate the Wnt/β-catenin pathway via linking Pygo2 PHD domain to β-catenin 9 and activating T-cell factor (TCF)/lymphoid-enhancing factor 1 (LEF) transcription 10. Pygo2 played an important role in transcription regulation, tissue differentiation and embryonic development 11, 12. Pygo2 is usually maintained at a low level in normal cells but has shown an abnormally high expression level in some cancers. Overexpression of Pygo2 acted as a driver for metastatic prostate cancer and esophageal squamous cell carcinoma by enhancing tumor growth and invasion 13, 14. Abnormal expression of Pygo2 also suggested a malignant phenotype in advanced lung cancer 15. Moreover, Pygo2 overexpression was associated with chemoresistance and poor prognosis in breast cancer and hepatocellular carcinoma 7, 16. Inhibition of Pygo2 expression increased the sensitivity of breast cancer cells to chemotherapeutic drugs and suppressed tumor growth 17. However, the expression level of Pygo2 in GC and the relationship between Pygo2 and MDR1 was undefined. In the present study, we detected the expression level of MDR1 and Pygo2 in GC and explored the functional relationship between them.

## Methods

### Materials

Cis-platinum (DDP) was obtained from the first affiliated hospital of Xiamen University. MDR1 (ABCB1, Cat# sc-55510) and β-actin (Cat# sc-47778) antibodies were purchased from Santa Cruz Biotechnology. Pygo2 (Cat# 11555-1-AP) antibody was obtained from Proteintech and TIAN pure Midi Plasmid Kit (Cat# DP107) from TIANGEN. TurboFect Transfection Reagent (Cat# R0531) was from Thermo Scientific. E.coli pLL3.7-hPygo2-KD (knock-down) and pGL3.3-control were a gift from Xiamen University School of Life Sciences.

### Cell culture and specimen collection

The GC cell lines, SGC7901 and SGC7901/DDP were cultured in DMEM medium with 10% fetal bovine serum and 1% penicillin/streptomycin. The GC tissues were collected from 40 GC patients using gastroscopy and surgery. All the GC patients were treated with platinum-based neoadjuvant chemotherapy, including combined with capecitabine, tegafur gimeracil oteracil potassium capsule (S-1), doxorubicin (ADM) and 5-fluorouracil (5-Fu). Importantly, all subjects were given written informed consent in accordance with the recommendations of Ethics and Scientific Committee of The First Affiliated Hospital of Xiamen University and the Declaration of Helsinki, the approved number from the Ethics committee of Xiamen University is 2019118.

### MTT assay

Cell viability was measured by using the MTT method. Briefly, SGC7901 and SGC7901/DDP cells (5×10^5^/well) were seeded in 96-well plates for 24 h, then treated with different concentrations of DDP for 24 h. Thereafter, cells were labeled with 20 μL MTT labeling reagent, and absorbance at 570 nm was determined with a microplate reader.

### The construction of drug resistance SGC7901/DDP

The SGC7901/DDP cell was constructed by treatment with increasing DDP concentration repeatedly and gradually. Firstly, SGC7901 cells were seeded at plates and treated with initial DDP concentration at 0.1 μg/mL for 24 h, then the medium was removed, and the cells were transferred to fresh medium. The above procedures were repeated, and DDP concentration was increased gradually until the cell apoptosis rate was above 90%. Cell morphology was observed by microscopy.

### Cell apoptosis assay

Cell apoptosis rate was determined by flow cytometry analysis using Annexin V-FITC Apoptosis Detection Kit (KEYGEN Biotech, Cat# KGA105-KGA108). 5×10^5^ cells were seeded in 6-well plates and treated with 0.4 μg/mL DDP for 24 h. The collected cells were washed by PBS twice and then labeled with 5 μL Annexin V-FITC and 5 μL Propidium iodide for 5 minutes at 25 ℃. The stained cells were analyzed by flow cytometry using CytExpert2.0 software.

### Western blot assay (WB)

Total protein extracts were prepared, and the protein concentration was measured as described previously 18. Generally, 15 μg protein was separated by 10% SDS-PAGE and transferred onto PVDF membrane, then blocked with 5% fat-free milk and incubated with primary antibodies and HRP- conjugated secondary antibodies. The protein levels were detected by using the Enhanced Chemiluminescent (ECL) Detection Kit (Boster, Cat# EK1001).

### Real-time quantitative PCR

Total RNAs were homogenized in Trizol and isolated, as described in our previous study 19. Then cDNAs were reversely transcribed by using PrimeScript^TM^ RT reagent kit with gDNA Eraser (Takara, Cat# RR047A). The expression levels of MDR1 and Pygo2 were detected using SYBR GREEN MIXTURE kit (Bio-Rad, Cat# 10000076382) and analyzed by comparing to β-actin. The primers are listed below: Pygo2, Forward Primer: 5'-GTTTGGGCTGTCCTGAAAGTCTG-3', Reserve Primer: 5'-ATAAGGGCGCCGAAAGTTGA-3'; MDR1, Forward Primer: 5'-AGCTCGTGCCCTTGTTAGACA-3', Reserve Primer: 5'-GTCCAGGGCTTCTTGGACAA-3'; β-actin, Forward Primer: 5'-CGAGCGGGAAATCGTGCGTGACATTAAGGAGA-3', Reserve Primer: 5'-CGTCATACTCCTGCTTGATCCACATCTGC-3'.

### Immunohistochemistry (IHC)

GC tissues were fixed and cut into slices, then processed and incubated with primary antibodies and probed with secondary antibody. The detailed protocol could refer to our previous work 19. Images were captured by using a Nikon microscope.

### pLL3.7- hPygo2-knock-down (KD) transfection

SGC7901/DDP cells were transfected with pGL3.3-Control and pLL3.7-hPygo2-KD plasmid using TurboFect Transfection Reagent following the manufacturer's instructions. Briefly, short hairpin targeted Pygo2 (Pygo2 shRNA) and the scramble control sequences (SCR shRNA) were designed based on hPygo2 sequences and further synthesized for the construction of plasmids 20. The detailed hPygo2 sequences could be referred to GenBank with accession number NW_925683. The primers were listed as follow: SCR shRNA, Forward Primer: 5'-GATCCCCGTGGTTTCATCGCATCTGCTTCAAGAGAGCAGATGCGATGAAACCACTTTTTA-3', Reserve Primer: 5'-AGCTTAAAAAGTGGTTTCATCGCATCTGCTCTCTTGAAGCAGATGCGATGAAACCACGGG-3'; Pygo2 shRNA, Forward Primer: 5'- GATCCCCTGTGAGGCCTCTTGTCAGAAATTCAAGAGATTTCTGACAAGAGGCCTCACATTTTTA-3', Reserve Primer: 5'-GGGACACTCCGGAGAACAGTCTTAAGTTCTCTAAAGACTGTTCTCCGGAGTGTAAAAATTCGA-3'. The lentiviral pLL3.7 vector (X-Y Biotechnology, Cat# XY2204) was used to express Pygo2 shRNA and pGL3.3 vector (Promega, Cat# E1741) served as a control, the plasmids were obtained by using TIAN pure Midi Plasmid Kit and the detailed procedures were described previously 21. The transient transfection was observed under a fluorescence microscope and verified by WB.

### Luciferase reporter analysis

The pGL6-MDR1 reporter plasmid, which contained the MDR1 promoter region was constructed in our lab and described in our previous study 22. The pGL6 reporter plasmid (318 bp) was designed as below: forward primer: 5′-CAGGGTACCAGTTGAAATGTCCCCAATGAT-3′, reverse primer: 5′-CCTAGATCTGGAAAGACCTAAAGGAAACGAAC-3′. Briefly, SGC7901/DDP cells were transfected with the pGL6-MDR1 reporter plasmid, and cell homogenate was lysed and collected for luciferase reporter analysis.

### Chromatin immunoprecipitation (ChIP) assay

The primers for ChIP on MDR1 were designed as described previously 22. MDR1 primers were listed as below: forward primer: 5′-GCGTTTCTCTACTTGCCCTTTC-3′, reverse primer: 5′-AGCCAATCAGCCTCACCACAG-3′. ChIP assay was performed by using a ChIP assay kit (Merck-Millipore, Cat# 17-371) per to the manufacturer's recommendations.

### Statistical analysis

The experiments such as MTT assay, WB and RT-PCR were performed in triplicate and data was expressed as the mean ± SD. Collective data was then analyzed using student's t-test and chi-square test. Correlation analysis was processed by using GraphPad Prism 7.0. P<0.05 was defined as the statistical significance and represented with *. *P<0.05, **P<0.01, ***P<0.001, ****P<0.0001.

## Results

### SGC7901/DDP was insensitive to chemotherapeutic drugs

**Figure 1:**
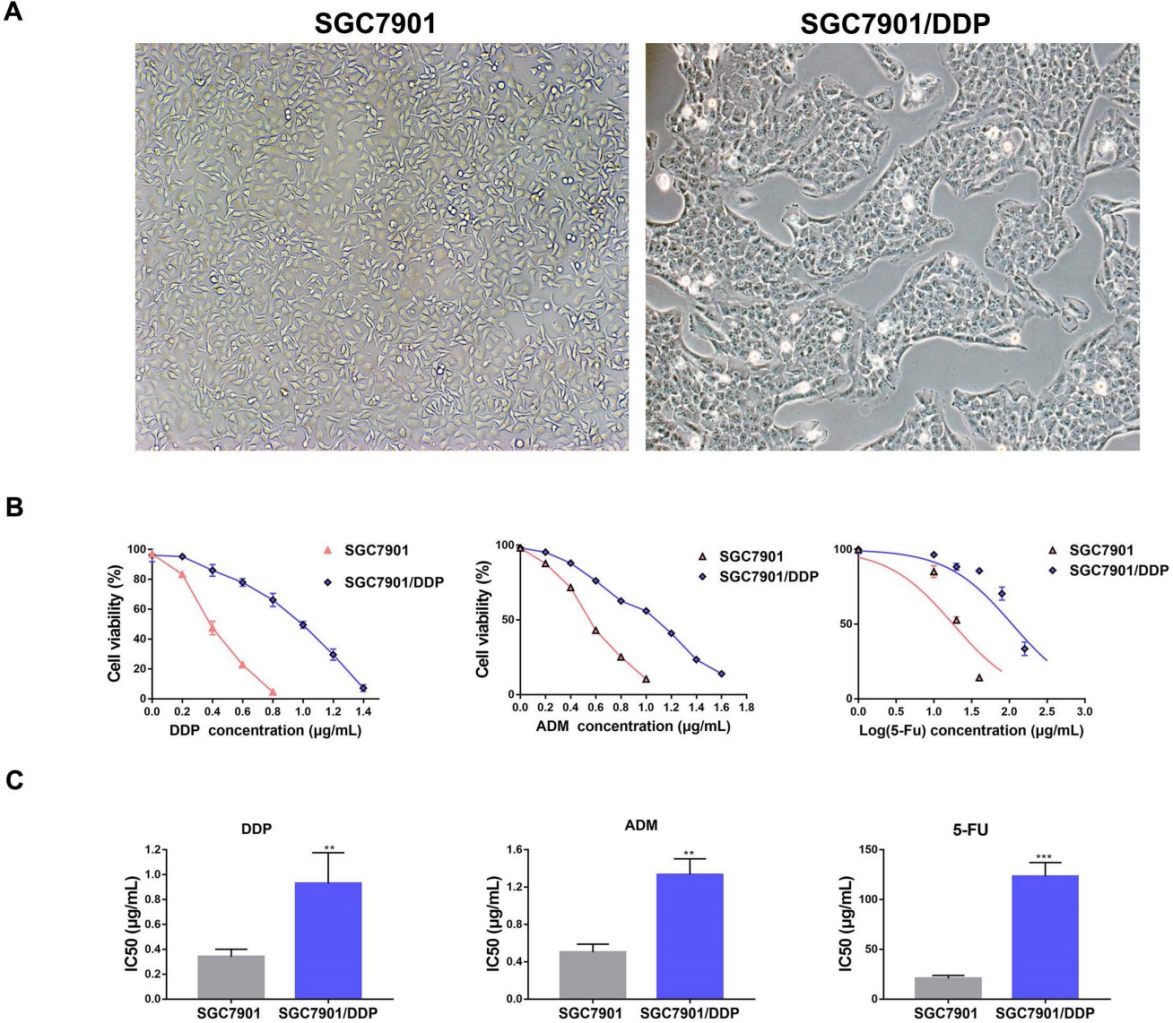
The construction of GC drug resistance cell SGC7901/DDP. **A.** SGC7901 and SGC7901/DDP was observed by using a microscopy and the images were captured, magnification: ×40. **B.** SGC7901 and SGC7901/DDP cells were treated with different concentrations of DDP, 5-Fu and ADM for 24 h and the cell viability was measured by MTT assay. **C.** IC50 of DDP, 5-Fu and ADM in SGC7901 and SGC7901/DDP. Each experiment was repeated three times, **P<0.01. ***P<0.001. DDP: cis-platinum; ADM: Adriamycin; 5-Fu: 5-fluorouracil.

We successfully constructed GC drug resistance cell SGC7901/DDP after six months utilizing the method described above. Comparing to SGC7901, SGC7901/DDP had a smaller cell volume, and its cell shape had changed from long spindle to short polygon. Furthermore, SGC7901/DDP had a slower breeding rate and showed monoclonal colony growth (**Fig. 1A**). SGC7901/DDP was insensitive to a low concentration of DDP when cells were exposed to different concentrations of DDP (**Fig. 1B**). The median IC50 value of SGC7901/DDP in 24 h was 0.89 μg/mL, which was significantly higher than SGC7901 with a median IC50 value in 24 h was 0.35 μg/mL (**Fig. 1C**). ADM and 5-Fu were also commonly prescribed to treat GC, we found that SGC7901/DDP was also insensitive to 5-Fu and ADM compared to SGC7901. The median IC50 values of 5-Fu and ADM in SGC7901/DDP for 24 h were 109.1 μg/mL and 1.3 μg/mL, which were higher than 20.5 μg/mL and 0.5 μg/mL in SGC7901 (**Fig. 1C**).

### High expression of Pygo2 and MDR1 in SGC7901/DDP and GC patients after chemotherapy

**Figure 2:**
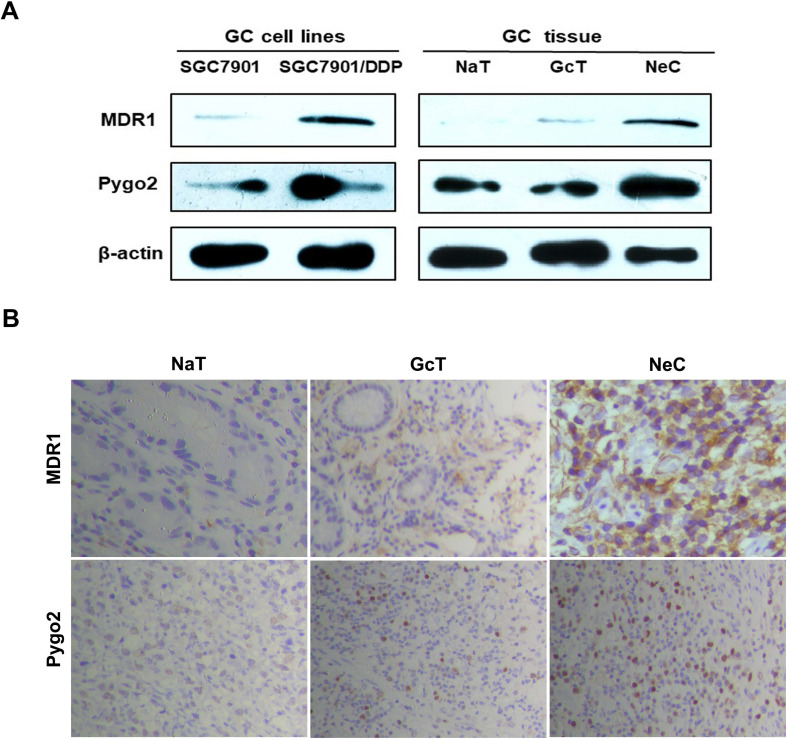
Pygo2 and MDR1 were overexpression in GC cell lines and GC patients after chemotherapy. **A.**The Pygo2 and MDR1 protein expression levels were detected by WB. **B.** The expression levels of Pygo2 and MDR1 in GC tissue measured by IHC, magnification: ×40. IHC: immunohistochemistry; NaT: normal-appearing tissue adjacent to GC, GcT: GC tissue; NeC: GC tissue after neoadjuvant chemotherapy.

**Figure 3:**
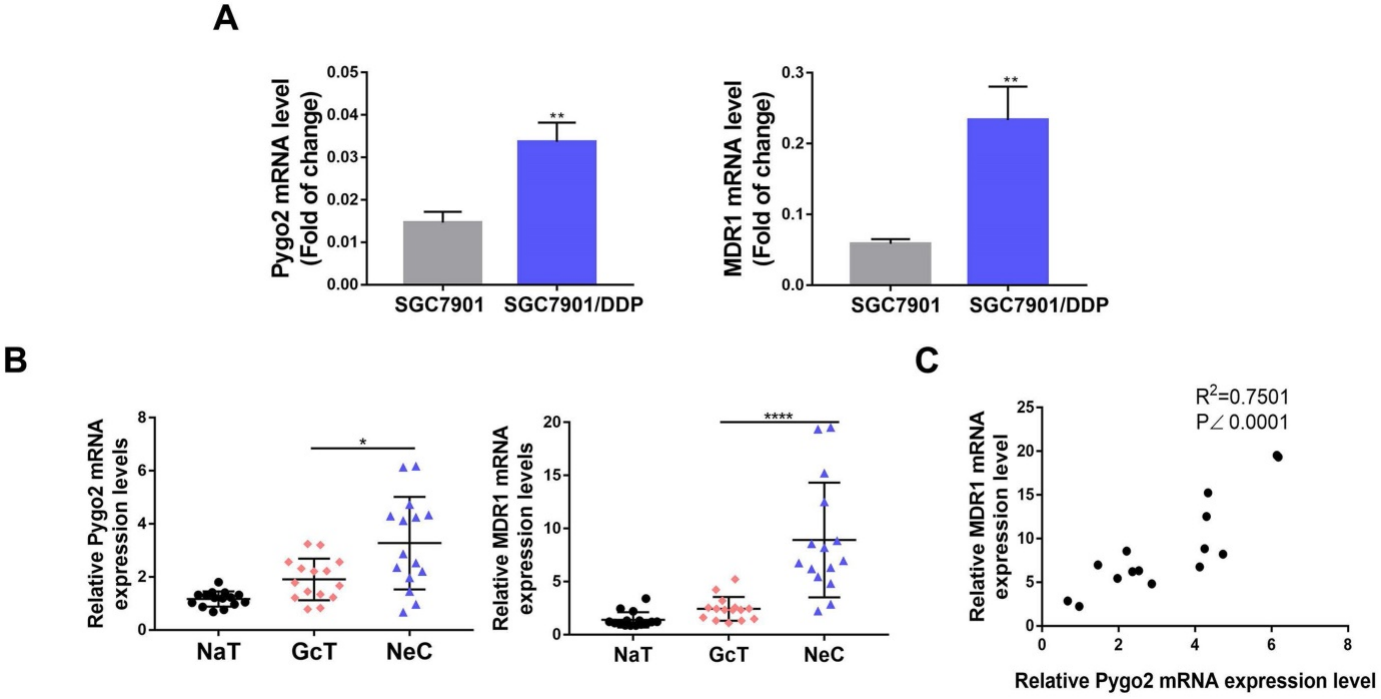
Pygo2 overexpression was strong corelated with MDR1. The Pygo2 and MDR1 RNA expression levels were detected by RT-PCR in GC cell lines (**A**) and GC tissues (**B**). **C.**The Pearson's R correlation was analyzed between Pygo2 and MDR1 expression levels in GC patients after chemotherapy. NaT: normal-appearing tissue adjacent to GC, GcT: GC tissue; NeC: GC tissue after neoadjuvant chemotherapy.

Firstly, we detected the protein and mRNA expression levels of Pygo2 and MDR1 in SGC7901/DDP by WB and RT-PCR. Our results showed that SGC7901/DDP had higher expression levels of Pygo2 and MDR1 compared with SGC7901 (**Fig. 2A and 3A**). Next, we analyzed Pygo2 and MDR1 expression levels in normal-appearing tissue adjacent to GC (NaT), GC tissue (GcT) and GC tissue after neoadjuvant chemotherapy (NeC). Our data revealed that MDR1 and Pygo2 was low or rarely expressed in NaT but were highly expressed in GcT and with highest expression levels in NeC (**Fig. 2A, Fig. 2B and 3B**).

### Pygo2 overexpression preceded MDR1 and Pygo2 correlated with MDR1

We analyzed 40 GC patients in the first affiliated hospital of Xiamen University who had undergone neoadjuvant DDP-based chemotherapy. Our results showed that 15% (6/40) of GC patients were Pygo2 overexpression positive, and 5% (2/40) were MDR1 overexpression positive pre-chemotherapy. While amongst the group, 62.5% (25/40) of GC patients were Pygo2 overexpression positive, and 45% (18/40) were MDR1 overexpression positive post-chemotherapy. The results indicated that Pygo2 overexpression might precede MDR1 (**Table 1**). Furthermore, analyzing the two genes across online data sets showed that a strong correlation between Pygo2 and MDR1 expression levels after chemotherapy in GC tissue (**Fig. 3C**).

### The knock-down of Pygo2 induced MDR1 downregulation and rendered GC cell sensitive to chemotherapeutic drugs

**Figure 4:**
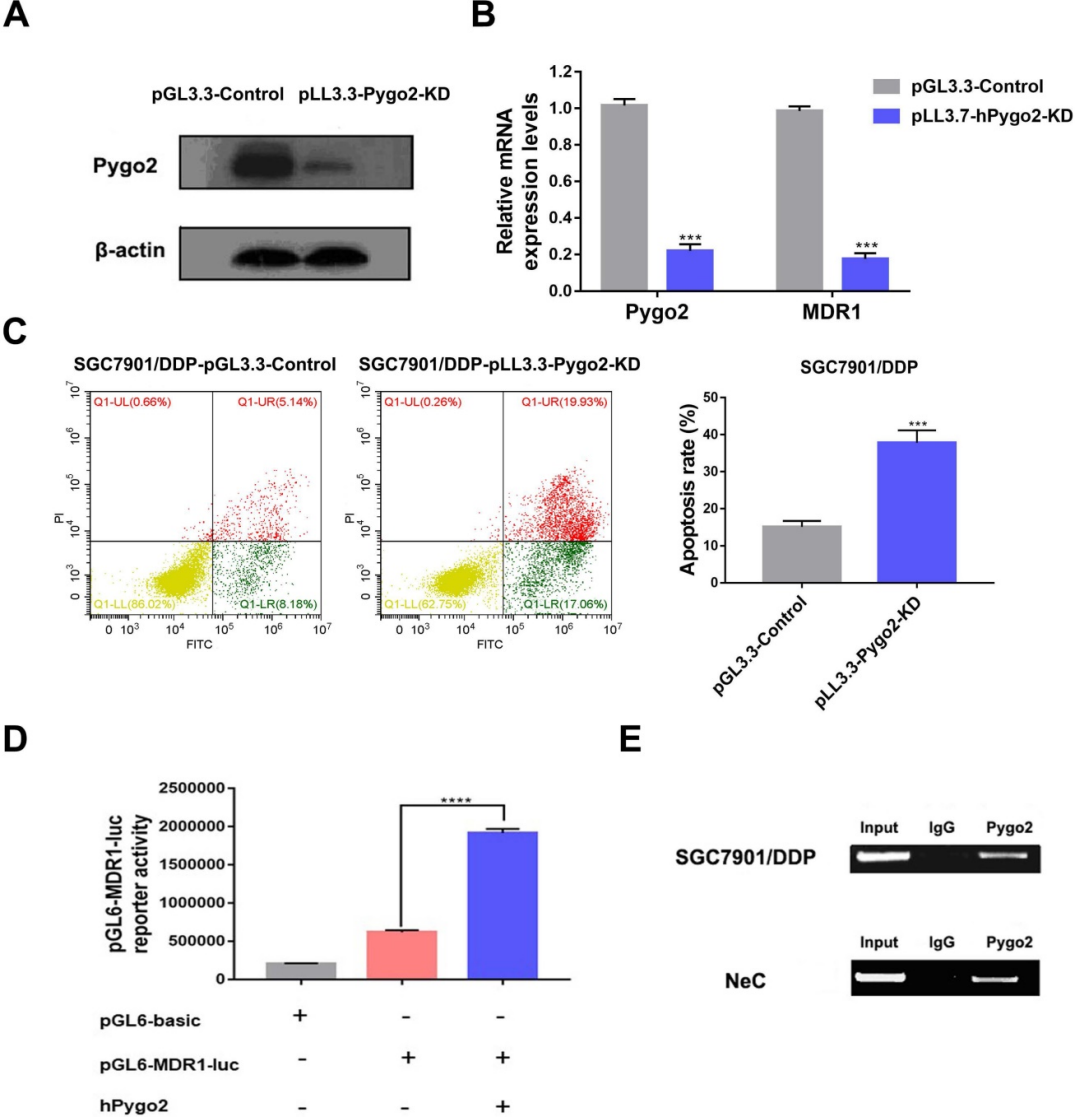
Pygo2 modulated MDR1 expression in GC. **A.** The knock-down of Pygo2 down was identified by WB after transfection. **B.** The expression levels of Pygo2 and MDR1 in SGC7901/DDP-pLL3.3-Pygo2-KD and SGC7901/DDP-pGL3.3. **C.** SGC7901/DDP-pLL3.3-Pygo2-KD and SGC7901/DDP-pGL3.3 were treated with 0.4 µg/mL DDP for 24 h, the apoptosis rate was analyzed by flow cytometry. **D.** SGC7901/DDP cells were transfected with the indicated plasmids and MDR1-reporter gene luciferase activity was measured. **E.** The interaction between Pygo2 and MDR1 promoter region using anti-Pygo2 antibody in SGC7901/DDP and GC tissue after neoadjuvant chemotherapy (Nec) by ChIP assay. ***P<0.001.

To clarify the relationship between Pygo2 and MDR1 in GC, we suppressed Pygo2 expression in SGC7901/DDP by transfecting pLL3.7-hPygo2-KD plasmid. We proceeded to perform WB to determine whether Pygo2 was successfully suppressed (**Fig. 4A**). The results suggested that when Pygo2 was inhibited, MDR1 expression was also downregulated (**Fig. 4B**). In addition, GC drug resistance cell SGC7901/DDP became sensitive to chemotherapeutic drugs when Pygo2 was knocked down (**Fig. 4C**).

### Pygo2 promoted MDR1 activation by interacting with MDR1

To illuminate the role of Pygo2 in the activation of MDR1, we performed luciferase reporter analysis. We found that Pygo2 could promote the activation of MDR1 promoter (**Fig. 4D**). Further mechanism analysis by ChIP assay verified the association of Pygo2 with the MDR1 promoter region (**Fig. 4E**). One other point worth emphasizing is our previous study also demonstrated Pygo2 could modulate MDR1 activation in breast cancer by directly binding to β-catenin, thus regulating the Wnt/β-catenin signaling pathway 7.

Taking into consideration the obtained results; we can conclude that the overexpression of Pygo2 promotes MDR1 activation and is involved in DDP drug resistance in GC.

## Discussion

Majority of GC patients are diagnosed at advanced stage disease as GC has a relatively low early detection and diagnosis rate. Chemotherapy is an important alternative therapeutic method for these patients. However, the development of MDR not only causes failure of the first-line treatment recommended by National Comprehensive Cancer Network (NCCN), but also results in the ineffectiveness of the second-and third-line solutions 23. Therefore, it is important to elucidate the mechanism of chemoresistance.

Studies exploring the mechanism of drug resistance are mainly divided into the following categories: alterations in drug efflux 24, dysfunction of DNA damage repair 25, disruption of apoptosis balance such as overexpression of anti-apoptosis proteins 26 and mutation of drug targets 27. One of the most studied mechanism is overexpression of MDR1 and multi-drug resistant associate protein (MRP), which serve as drug efflux transporters leading to a decrease in intracellular drug concentration 28. MDR1 overexpression was identified in GC cell lines and primary GC tissue in previous studies 29. Additionally, MDR1 overexpression was negatively associated with chemosensitivity 30. Studies on MDR transporter inhibitors have been flourishing, but with little success 31. Hence, it was essential to identify novel MDR biomarkers and drug targets.

Pygo2 is a novel effector protein, downstream of the Wnt/β-catenin signaling pathway, that acts as a co-activator with β-catenin to promote β-catenin/LEF/TCF activation 32. Abnormal expression of Pygo2 has been reported in several cancers such as breast cancer, glioma, esophageal squamous cell carcinoma, hepatocellular carcinoma and prostate cancer 7, 13, 14, 16, 33-35. Furthermore, Pygo2 overexpression was associated with tumor initiation and progression, malignant phenotype, and poor prognosis 13, 15, 16, 34. Inhibition of Pygo2 suppressed tumor growth, invasion, and epithelial-mesenchymal transition (EMT) 17. Our previous study showed that Pygo2 was associated with chemoresistance in breast cancer by activating MDR1 7. MDR1 was the downstream target of the Wnt/β-catenin signaling pathway, and it was upregulated when the Wnt/β-catenin was activated 36. Pygo2 not only promoted Wnt/β-catenin activation but also modulated Wnt/β-catenin activity in tissue-and gene-dependent manner 11, 37, 38. Thus, whether Pygo2 regulates MDR1 in GC or not still needs further study.

These recent findings verified that Pygo2 and MDR1 were overexpressed in GC, especially in GC patients after chemotherapy. Pygo2 overexpression precedes MDR1 and Pygo2 strongly correlated with MDR1 in GC patients who underwent neoadjuvant chemotherapy. The Knock-down of Pygo2 could restore the drug-resistant cell SGC/7901 sensitivity to chemotherapeutic drugs. Further mechanism analysis indicated that Pygo2 could promote MDR1 activation by binding to the MDR1 promoter region. These results demonstrated that Pygo2 might be a novel biomarker for monitoring drug resistance by upregulating MDR1. This study also provided a potential therapeutic target in the treatment of cancers where Pygo2 is overexpressed.

In conclusion, Pygo2 and MDR1 were overexpressed in GC patients after chemotherapy. Pygo2 played a pivotal role in drug resistance in GC by promoting MDR1 activation. Pygo2 may be a novel biomarker for monitoring drug resistance and a potential therapeutic target for cancer treatment in the future.

## Figures and Tables

**Table 1:** The expression status of Pygo2 and MDR1 in GC patient pre-and post-operation

Group		Pre chemotherapy (Gastroscopy)	Post chemotherapy (Postoperative biopsy)	χ^2^-test
				*P* value
Pygo2	Overexpression	6 (15%)	25 (62.5%)	<0.0001
	None overexpression	34 (85%)	15 (37.5%)	
MDR1	Overexpression	2 (5%)	18 (45%)	<0.0001
	None overexpression	38 (95%)	22 (55%)	
